# Efficacy and safety of anagrelide as a first‐line drug in cytoreductive treatment‐naïve essential thrombocythemia patients in a real‐world setting

**DOI:** 10.1111/ejh.13265

**Published:** 2019-06-17

**Authors:** Tomoki Ito, Yoshinori Hashimoto, Yasuhiro Tanaka, Aya Nakaya, Shinya Fujita, Atsushi Satake, Takahisa Nakanishi, Akiko Konishi, Masaaki Hotta, Hideaki Yoshimura, Kazuyoshi Ishii, Akiko Hashimoto, Toshinori Kondo, Hiromi Omura, Isaku Shinzato, Takayuki Tanaka, Shosaku Nomura

**Affiliations:** ^1^ First Department of Internal Medicine Kansai Medical University Hirakata Japan; ^2^ Department of Hematology Tottori Prefectural Central Hospital Tottori Japan; ^3^ Department of Hematology and Clinical Immunology Kobe City Nishi‐Kobe Medical Center Kobe Japan; ^4^ Department of Hematology Kawasaki Medical School Kurashiki Japan

**Keywords:** anagrelide, essential thrombocythemia, first‐line, treatment

## Abstract

**Objective:**

This study aimed to retrospectively assess the efficacy and safety of anagrelide in cytoreduction therapy‐naïve essential thrombocythemia (ET) patients in a real‐world setting.

**Method:**

Data from 53 ET patients who received anagrelide as a first‐line therapy were reviewed for patient characteristics, antiplatelet status, cytoreduction status, therapeutic effects, adverse events, thrombohemorrhagic event development, progression to myelofibrosis or acute leukemia, and cause of death.

**Results:**

The rate of achieving a platelet count of <600 × 10^9^/L during anagrelide monotherapy was 83.0%. Adverse events occurred in 32 of 53 patients, and tended to be slightly more severe in patients with cardiac failure; however, they were mostly tolerable. The therapeutic effect of anagrelide was consistent, regardless of genetic mutation profiles. The incidence of anemia as an adverse event was significantly higher in the *CALR* mutation‐positive group. Favorable platelet counts were also achieved in patients for whom hydroxyurea was introduced as a replacement for anagrelide or in addition to anagrelide because of unresponsiveness or intolerance to treatment.

**Conclusion:**

In Japanese cytoreduction therapy‐naïve ET patients, anagrelide administration as a first‐line therapy demonstrated favorable effects in reducing platelet counts, and its safety profile that was generally consistent with those in previous reports.

## 1 INTRODUCTION

Essential thrombocythemia (ET) is a myeloproliferative neoplasm (MPN), that is, characterized by a sustained platelet increase in the peripheral blood and anomalous megakaryocyte growth in bone marrow biopsy.^1^ Treatment goals comprise prevention of thrombohemorrhagic events (THEs), progression to myelofibrosis (MF) or acute leukemia (AL), and the onset of secondary malignancies. Many guidelines recommend antiplatelet and cytoreduction therapy for patients who are at high risk of thrombosis 2, 3, 4; in particular, the recently revised European LeukemiaNet (ELN) recommendations that recommend hydroxyurea and interferon‐α as first‐line therapies for cytoreduction therapies. If hydroxyurea is ineffective or cannot be tolerated, anagrelide and interferon‐α are recommended as second‐line therapies.^2^ Anagrelide is a unique quinazoline derivative, that is, used for treatment of thrombocytopenia, although it was initially developed as an inhibitor of platelet aggregation.^5^ In 1997, anagrelide was approved in the United States as a therapeutic agent for thrombocytosis associated with MPN; in 2004, it was approved in Europe for the treatment of high‐risk ET patients. However, because of the results of a primary thrombocythemia‐1 (PT‐1) trial published in 2005,^6^ anagrelide remains classified as a second‐line therapy in Europe,^2^ and it is classified as less than second‐line therapy in the United States.^7^ Based on the results of the ANAHYDRET Study, which showed the non‐inferiority of anagrelide to hydroxyurea,^8^ as well as the results of a phase III clinical trial in Japanese patients (published in 2013),^9^ anagrelide was approved in Japan as a first‐line therapy for ET patients in 2014. In Europe, there have been concerns regarding a risk of leukemogenesis, based on the results of a large‐scale joint observational study conducted in 13 European countries (Evaluation of Anagrelide Efficacy and Long‐term Safety [EXELS] Study 10, 11, 12, 13; thus, anagrelide is mainly used in young ET patients, which has led to a gap in therapeutic agents between European countries and the United States. The discussion of anagrelide efficacy and safety is ongoing and a consensus has not been reached. The present study retrospectively examined the efficacy and safety of anagrelide in cytoreduction therapy‐naïve ET patients in Japan where anagrelide is approved as a first‐line therapy for ET treatment.

## 2 PATIENTS AND METHODS

Fifty‐three ET patients (31 at Kansai Medical University, 16 at Tottori Prefectural Central Hospital, and six at Kobe City Nishi‐Kobe Medical Center) were included in this retrospective study of anagrelide as a first‐line therapy. The attending physicians explained the benefits and limitations of both hydroxyurea and anagrelide to patients in clinical practice; patients were included consecutively in our cohort when they selected administration of anagrelide. Based on medical records, the following data were examined: patient characteristics including driver gene mutations, history of THEs, presence or absence of cardiovascular risk factors (eg, defined as diabetes mellitus, hypertension, high low‐density lipoprotein [LDL] cholesterolemia, hyperlipidemia, and/or smoking), treatment statuses of antiplatelet and anagrelide therapies, presence or absence of cytoreduction combination therapy, therapeutic effects, adverse events, THEs after diagnosis or the initiation of cytoreduction therapy with anagrelide, progression to MF or AL, onset of secondary malignancies, and cause of death. This study was approved by the ethics committees at Kansai Medical University, Tottori Prefectural Central Hospital, and Kobe City Nishi‐Kobe Medical Center.

### 2.1 Treatment

Anagrelide was started at a dose of 0.5 or 1.0 mg/d and was continued at least for 1 week based on the instructions for use. The dose was then increased until the minimum effective dose was reached; the maximum increase was up to 0.5 mg/d/wk, and the maximum daily dose was ≤5 mg/d. If anagrelide monotherapy was ineffective or if a patient showed intolerance to an increased dose, treatment was switched to hydroxyurea alone or hydroxyurea combined with anagrelide at the discretion of the attending physicians. We administered antiplatelet drugs to patients with a history of thrombosis, patients with cardiovascular risk factors, and patients with *JAK2V617F* mutation; if a patient refused antiplatelet drugs, we did not administer them.

### 2.2 Definition

The World Health Organization (WHO) classification 2008^14^ and 2016^1^ classifications were used as a diagnostic criteria for ET. The thrombosis risk category was stratified in accordance with the following previously reported major risk classifications: conventional risk classification,^15^ International Prognostic Score of Thrombosis for Essential Thrombocythemia (IPSET‐thrombosis),^16^ and revised IPSET‐thrombosis.^17^ With respect to THEs, thrombotic events were defined as stroke, transient ischemic attack (TIA), myocardial infarction, angina pectoris, peripheral arterial occlusive disease, erythromelalgia, deep vein thrombosis, and pulmonary embolism; hemorrhagic events were defined as cerebral hemorrhage, gastrointestinal hemorrhage, hematuria, and mucosal hemorrhage. The therapeutic effect of cytoreduction therapy was evaluated based on the ELN criteria.^15^ Complete response (CR) was defined as a platelet count of ≤400 × 10^9^/L, no disease‐related symptoms, and normal spleen size on imaging analysis, and white blood cell (WBC) count of ≤10 × 10^9^/L. Partial response (PR) was defined as an inability to meet the criteria for CR, with a platelet count of ≤600 × 10^9^/L or reduction of >50% from baseline. Adverse events were classified using the Common Terminology Criteria for Adverse events (CTCAE) Version 4.0. Secondary malignancies that occurred during the follow‐up period were defined as new malignancies, regardless of drug use. For MPN gene mutation analysis,^18^, ^19^, ^20^ polymorphonuclear leukocytes were isolated from blood samples. The presence or absence of *JAK2V617F* mutations and *MPL‐W515L/K* mutations was assessed using the DNA extraction and allele‐specific polymerase chain reaction (PCR) methods. With respect to the exon 9 region in *CALR* genes, the presence or absence of a mutation was confirmed using PCR or the direct sequencing method.

### 2.3 Statistical analysis

Demographic information for each patient was recorded; this included the patient's background information, treatment status, and event occurrences. Fisher's exact test was used for nominal variables, and the Mann‐Whitney U test was used for continuous variables. For all statistical analyses of effective variables, two‐tailed tests were performed, and *P*‐values <0.05 were considered to be statistically significant. EZR statistical analysis software (Jichi Medical University, Saitama, Japan) was used.^21^

## 3 RESULTS

Table 1 shows the background information for the 53 patients (22 men and 31 women) included in this study. Driver gene mutations included *JAK2V617F* mutations (n = 34), *CALR* mutations (n = 11 [type 1, n = 8; type 2, n = 2; and other, n = 1]), and *MPL* mutations (n = 1); some patients were negative for all of the above three driver gene mutations together (triple negative; n = 7). Seventeen patients had a history of thrombosis (thrombotic events that occurred before diagnosis of ET). Cardiovascular risk factors were diabetes mellitus (n = 8), hypertension (n = 16), high LDL cholesterolemia (n = 14), hypertriglyceridemia (n = 6), and smoking (n = 6). Twenty‐nine patients had at least one of the above cardiovascular risk factors. Six patients had cardiac failure (all of them were class I, based on the New York Heart Association classification). Based on the conventional thrombotic risk classification, 12 patients were low risk and 41 patients were high risk at the time of diagnosis. Before the initiation of anagrelide therapy, four patients were ≥60 years of age; based on this age, the classifications were modified as follows: Eight patients were low risk and 45 patients were high risk. Among the low‐risk patients, three had a platelet count of ≥1000 × 10^9^/L before starting anagrelide, and the other four were *JAK2V617F* mutation‐positive patients. Based on the IPSET‐thrombosis score, there were 12 low‐risk patients, six intermediate‐risk patients, and 35 high‐risk patients at the time of diagnosis. Based on the revised IPSET‐thrombosis score, there were four very low‐risk patients, nine low‐risk patients, 11 intermediate‐risk patients, and 29 high‐risk patients at the time of diagnosis. An antiplatelet agent was used in 28 patients; these patients had a history of thrombosis, cardiovascular risk factors, or *JAK2V617F* gene mutations.

**Table 1:** Main characteristics of 53 patients with ET

Patients characteristics	Total (n = 53)
Age at diagnosis, median (range)	67.0 (21‐93)
Male, n (%)	22 (41.5)
Female, n (%)	31 (58.5)
WBC, median; ×10^9^/L (range)	9.5 (5.7‐20.5)
Neutrophil rate, median; % (range)	71.4 (54.7‐87.0)
Hb, median; g/dL (range)	14.2 (8.6‐19.0)
Plt, median; ×10^9^/L (range)	913 (514‐2453)
LDH, median IU/L (range)	237 (171‐631)
*JAK2* gene mutation, n (%)	34 (64.2)
*CALR* gene mutation, n (%)	11 (20.8)
*MPL* gene mutation, n (%)	1 (1.9)
Triple‐negative, n (%)	7 (13.2)
History of thrombosis, n (%)	17 (32.1)
Cardiovascular risk factors, n (%)	29 (54.7)
Cardiac failure, n (%)	6 (11.3)
Antiplatelet medications, n (%)	28 (52.8)

**Figure 1:**
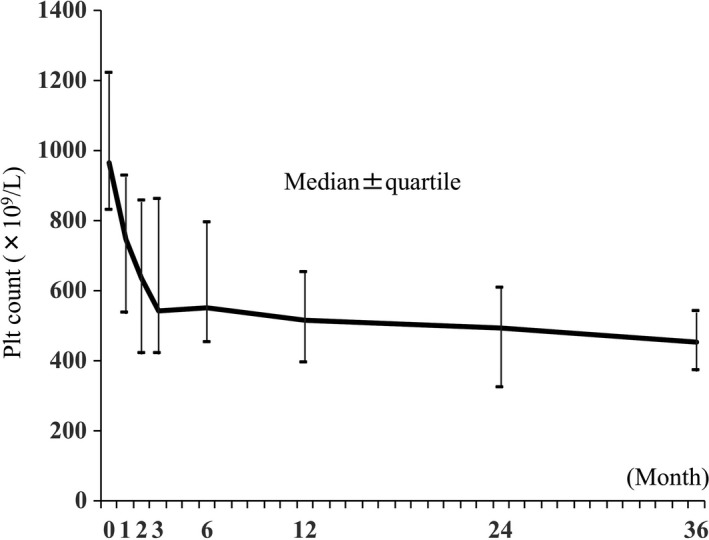
Median platelet count before and after the initiation of anagrelide therapy. Administration of anagrelide as a first‐line therapy demonstrated favorable effects in reducing platelet counts. The median platelet counts immediately before and at 1, 2, 3, 6, 12, 24, and 36 months after the initiation of anagrelide therapy were 965 × 10^9^/L, 747 × 10^9^/L, 635 × 10^9^/L, 542 × 10^9^/L, 551 × 10^9^/L, 514 × 10^9^/L, 495 × 10^9^/L, and 453 × 10^9^/L, respectively

The median duration of anagrelide therapy was 642 days (range, 43‐1219 days) (Table 2). The median daily dose was 1.44 mg/d (range, 0.53‐2.78 mg/d). There were 44 patients who achieved a platelet count of <600 × 10^9^/L during anagrelide monotherapy, which was achieved in a median of 53 days. The best overall response was a CR (n = 27; 50.9%), followed by PR (n = 18; 34%), and no response (NR; n = 8; 15.1%) (Table 2). Overall, hydroxyurea was used in 17 patients (32.1%) who exhibited a lack of efficacy or intolerance to anagrelide monotherapy. Eight of these 17 patients were switched from anagrelide to hydroxyurea because of adverse events (n = 4), reduced efficacy after successful response to anagrelide (n = 3), or thrombotic events (n = 1). Nine of these 17 patients were received hydroxyurea in addition to anagrelide because of insufficient efficacy of anagrelide monotherapy, at the discretion of attending physicians. The median platelet counts immediately before and after the initiation of anagrelide therapy are shown in Figure 1; patients in our cohort had a platelet response as a result of anagrelide‐based first‐line therapy, which was comparable to the thrombocytopenic effect of the ANAHYDRET Study.^8^

**Table 2:** Details of treatment with anagrelide, response, and adverse events

Treatment, response, and AEs	Total (n = 53)
Duration of anagrelide therapy, days	
Mean (SD)	656 (378)
Median (range)	642 (43‐1219)
Daily dose, mg/d	
Mean (SD)	1.46 (0.48)
Median (range)	1.44 (0.53‐2.78)
Response	
Number of achieving a Plt count <600 × 10^9^/L, n (%)	44 (83.0)
Complete response, n (%)	27 (50.9)
Partial response, n (%)	18 (34.0)
No response, n (%)	8 (15.1)
AEs (≥5% of patients)	
Palpitations, n (%)	14 (26.4)
Headache, n (%)	11 (20.8)
Anemia, n (%)	10 (18.9)
Diarrhea, n (%)	4 (7.5)
Cardiac failure, n (%)	3 (5.7)
AEs (grade 3)	
Anemia, n (%)	2 (3.8)
Cardiac failure, n (%)	2 (3.8)

Abbreviations: AE: adverse event; SD: standard deviation.

The median follow‐up period was 4.1 years and 32 of 53 patients had treatment‐related adverse events; 47 adverse events comprised 43 grade 1 or 2 events and four grade 3 events (Table 2). The most common adverse events were palpitations (n = 14; 26.4%), headache (n = 11; 20.8%), anemia (n = 10; 18.9%), diarrhea (n = 4; 7.5%), and cardiac failure (n = 3; 5.7%). Grade 3 adverse events consisted of anemia (n = 2; 3.8%) and cardiac failure (n = 2; 3.8%) (Table 2). With the exception of one patient with grade 3 anemia, the other three patients switched from anagrelide to hydroxyurea because of adverse events. Among the six patients with cardiac failure, five patients had cardiac adverse events: two had grade 3 cardiac failure, one had grade 3 anemia, one had grade 2 anemia, and one had grade 1 lower leg edema.

During the follow‐up period, 12 patients (22.6%) had THEs, eight patients had thrombotic events (15.1% [3.7/100 patient years]), (ie, cerebral infarction [n = 3], TIA [n = 1], myocardial infarction [n = 3], and angina [n = 1]), and four patients had hemorrhagic events (7.5% [1.8/100 patient years]) (ie, hematuria [n = 1], bloody sputum [n = 1], and epistaxis [n = 2]) (Table 3). The median time between the initiation of anagrelide therapy and event onset in patients with thrombosis events was 241.5 days, and values at the time of onset were as follows: median WBC count, 11.5 × 10^9^/L; median neutrophil rate, 81.2%; median Hb level, 13.0 g/dL; and median platelet count, 709 × 10^9^/L. Similarly, the median time between the initiation of anagrelide therapy and event onset in patients with hemorrhagic events was 547.5 days, and values at time of onset were as follows: median WBC count, 12.1 × 10^9^/L; median neutrophil count, 81.2%; median Hb level, 13.0 g/dL; and median platelet count, 449 × 10^9^/L. All three patients with transformation had MF. The times between diagnosis and transformation were 2.0, 1.7, and 1.2 years, whereas the times between the initiation of anagrelide therapy and transformation were 2.0, 1.6, and 0.8 years, respectively. No patient developed secondary malignancies. One patient with a history of cerebral infarction died of aspiration pneumonia.

**Table 3:** Development of THEs and transformation during anagrelide therapy

Details of events	Total (n = 53)
THEs, n (%)	12 (22.6)
Thrombotic events, n (%)	8 (15.1)
Time between the start of anagrelide therapy and event onset, median (range)	241.5(54‐914)
WBC at thrombosis, median; ×10^9^/L (range)	11.5 (7.5‐38.4)
Neutrophil rate at thrombosis, median; % (range)	81.2 (62.8‐89.0)
Hb at thrombosis, median; g/dL (range)	13.0 (11.5‐15.0)
Plt at thrombosis, median; ×10^9^/L (range)	709 (327‐1116)
Hemorrhagic events, n (%)	4 (7.5)
Time between the initiation of anagrelide therapy and event onset, median (range)	547.5(291‐1204)
WBC at hemorrhage, median; ×10^9^/L (range)	12.1 (6.7‐26.1)
Neutrophil rate at hemorrhage, median; % (range)	81.2 (64.5‐85.6)
Hb at hemorrhage, median; g/dL (range)	13.0 (9.8‐12.2)
Plt at hemorrhage, median; ×10^9^/L (range)	449 (174‐752)
Transformation, n (%)	3 (5.7)
MF, n (%)	3 (5.7)

Abbreviations: THEs: thrombohemorrhagic events; MF: myelofibrosis.

Table 4 shows the characteristics of ET patients who were *JAK2V617F* mutation‐positive (*JAK2‐*ET; n = 34; 64.2%) and *CALR* mutation‐positive (*CALR‐*ET; n = 11; 20.8%) groups. There were no significant differences in sex or age between the two gene mutation groups; however, the median WBC count, median neutrophil rate, and median Hb levels were significantly higher in the *JAK2*‐ET than in the *CALR‐*ET group. There was a trend indicative of higher median platelet counts were observed in the *CALR‐*ET group, but there was no significant difference between the two groups. Six patients in the *JAK2*‐ET group, but none in the *CALR*‐ET group, experienced thrombotic events during the course of therapy. The median duration of anagrelide therapy and median daily dose in the *JAK2*‐ET group were 698 days and 1.45 mg/d, respectively, whereas they were 666 days and 1.37 mg/d, respectively, in the *CALR*‐ET group. In the *JAK2*‐ET group, 27 patients (79.4%) achieved a platelet count of <600 × 10^9^/L during anagrelide monotherapy, which occurred within a median of 49 days. The best overall response was CR (50%), followed by PR (29.4%) and NR (20.6%). In contrast, in the *CALR*‐ET group, 10 patients (90.9%) achieved a platelet count of <600 × 10^9^/L during anagrelide monotherapy, which occurred within a median of 60 days. The best overall response was CR (36.4%), followed by PR (63.6%) and NR (0%). Changes in the WBC count, Hb level, and platelet count in both groups before and after anagrelide therapy are shown in Table S1. The median WBC count before and at any time point after the initiation of anagrelide therapy (1, 2, 3, 6, 12, 24, and 36 months) was significantly higher in the *JAK2*‐ET group than in the *CALR*‐ET group. Similarly, the median Hb level immediately before and at 1, 2, and 3 months after the initiation of anagrelide therapy was significantly higher in the *JAK2*‐ET group than in the *CALR*‐ET group. There were no significant differences in platelet count between the two groups at any time point.

**Table 4:** Characteristics of ET patients with *JAK2V617F* and *CALR* gene mutation

Variable	Mutation profiles		*P* value
	*JAK2V617F*	*CALR*	
Number of patients, n (%)	34 (64.2)	11 (20.8)	
Female, n (%)	21 (61.8)	6 (54.5)	0.732
Age at diagnosis, median (range)	67 (21‐93)	69 (43‐81)	0.663
WBC, median; ×10^9^/L (range)	10.3 (5.7‐18.0)	8.5 (5.7‐11.1)	0.004
Neutrophil rate, median; % (range)	74.3 (58.1‐87.0)	67.7 (56.5‐77.0)	0.026
Hb, median; g/dL (range)	14.3 (9.6‐19.0)	13.6 (8.6‐14.7)	0.014
Plt, median; ×10^9^/L (range)	868 (514‐1636)	967 (605‐1452)	0.144
LDH, median IU/L (range)	228 (171‐589)	261 (192‐373)	0.369
History of thrombosis, n (%)	13/34 (38.2%)	3/11 (27.3%)	0.720
Thrombosis after diagnosis, n (%)	6/34 (17.6)	0/11	0.311
Duration of anagrelide therapy, median; days (range)	698 (129‐1219)	666 (320‐1212)	0.367
Daily dose of anagrelide, median; mg/d (range)	1.45 (0.53‐2.78)	1.37 (0.93‐1.88)	0.517
Switch from anagrelide to hydroxyurea	7/34 (20.6%)	0/11	0.168
Addition of hydroxyurea to anagrelide	7/34 (20.6%)	2/11 (18.2%)	>0.999
Number of achieving a Plt count <600 × 10^9^/L, n (%)	27 (79.4)	10 (90.9)	0.657
Complete response, n (%)	17 (50.0)	4 (36.4)	0.503
Partial response, n (%)	10 (29.4)	7 (63.6)	0.0721
No response, n (%)	7 (20.6)	0 (0.0)	0.168

The rate of change was calculated, as shown in Table S2. There were no significant differences in the pre‐ and post‐anagrelide therapy trajectories (from baseline) of the median WBC counts, Hb levels, or platelet counts between the *JAK2*‐ET and *CALR*‐ET groups. In both groups, the respective median Hb levels and platelet counts at 24 months after the initiation of anagrelide were approximately 10% and 50% lower than at baseline values. This is potentially because the *JAK2*‐ET group included seven patients (20.6%) who switched from anagrelide to hydroxyurea, as well as seven patients (20.6%) who received hydroxyurea in combination with anagrelide (20 patients [58.8%] received anagrelide monotherapy); in contrast, the *CALR*‐ET group included two patients (18.2%) who received hydroxyurea in combination with anagrelide (nine patients [81.8%] received anagrelide monotherapy) (Table 4). Among patients who received anagrelide monotherapy, the pre‐ and post‐anagrelide therapy trajectories (from baseline) of the median WBC counts, Hb levels, and platelet counts for the *JAK2*‐ET (n = 20) and *CALR*‐ET (n = 9) groups showed similar reduction rates (Table S3). Regarding treatment‐related adverse events, the number of adverse events (anemia) was significantly higher in the *CALR*‐ET group (five patients [45.5%]) compared with the number of such events in the *JAK2*‐ET group (five patients [14.7%]; *P* = 0.037).

**Figure 2:**
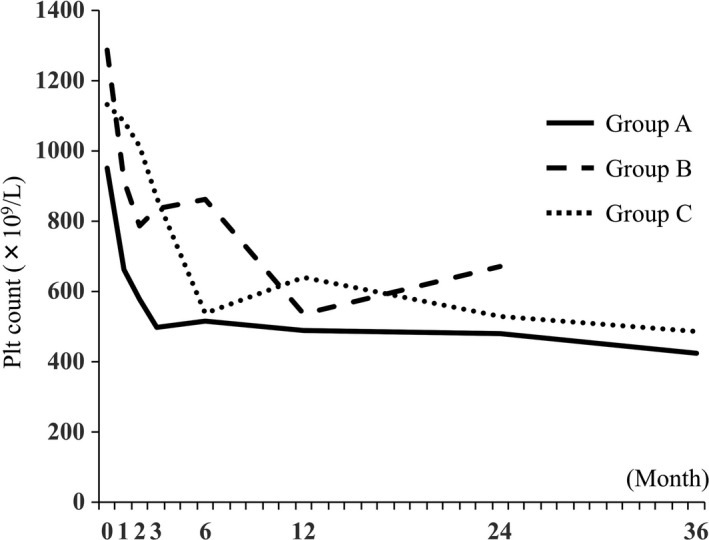
Median platelet count before and after treatment in each of the three groups. Group A is consisted of those who received anagrelide monotherapy (n = 36); group B is consisted of those who switched from anagrelide to hydroxyurea (n = 8); group C is consisted of those who received hydroxyurea in combination with anagrelide (n = 9). In group A, the median platelet counts immediately before and at 1, 2, 3, 6, 12, 24, and 36 months after the initiation of anagrelide therapy were 951 × 10^9^/L, 661 × 10^9^/L, 578 × 10^9^/L, 497 × 10^9^/L, 517 × 10^9^/L, 490 × 10^9^/L, 480 × 10^9^/L, and 424 × 10^9^/L, respectively. Likewise, in group B they were 1287 × 10^9^/L, 916 × 10^9^/L, 787 × 10^9^/L, 837 × 10^9^/L, 863 × 10^9^/L, 536 × 10^9^/L, and 671 × 10^9^/L, respectively, and in group C there were 1132 × 10^9^/L, 1083 × 10^9^/L, 1011 × 10^9^/L, 866 × 10^9^/L, 537 × 10^9^/L, 639 × 10^9^/L, 530 × 10^9^/L, and 486 × 10^9^/L, respectively

As described above, 36 patients (67.9%) received anagrelide monotherapy (A), eight patients (15.1%) switched from anagrelide to hydroxyurea (B), and nine patients (17.0%) received hydroxyurea in combination with anagrelide (C). Changes in the median platelet count before and after anagrelide therapy in each of the three groups are shown in Figure 2. Anagrelide monotherapy (group A) showed a good thrombocytopenic effect; additionally, combination therapy (group C) and, to a lesser extent, switched therapy from anagrelide to hydroxyurea (group B), showed good thrombocytopenic effects.

Twelve patients (22.6%) discontinued anagrelide monotherapy because of adverse events (n = 5), insufficient efficacy (n = 4), thrombotic events (n = 1), progression to MF (n = 1), or death (n = 1).

## 4 DISCUSSION

The largest study of anagrelide as a first‐line therapy in ET patients was the ANAHYDRET Study,^8^ while others have been much smaller.^22^, ^23^ In the present study, we have reported the efficacy and safety results of anagrelide based on actual clinical data in Japan where the drug is approved as a first‐line therapy. This represents a rare report of the efficacy of switching from anagrelide as a first‐line therapy to hydroxyurea, as well as the efficacy of the addition of hydroxyurea to the anagrelide therapy regimen. In the present study, anagrelide showed thrombocytopenic effects comparable to those of the ANAHYDRET Study,^8^ a phase III clinical trial in Japanese patients,^9^ and a phase I/II clinical trial in untreated patients.^24^ Our cohort is unique because the median daily anagrelide dose (1.44 mg/d) was comparatively lower than that in the studies where it was used as a first‐line therapy.^8^, ^22^, ^23^ Moreover, this dose was similar to that used in anagrelide and hydroxyurea combination therapy by Gugliotta et al^25^ In the present study, the rate of adverse events was similar to, or slightly lower than, that of previous studies^8^, ^9^, ^24^; this may be due to the low daily doses of anagrelide. Notably, anagrelide was administered to some patients with cardiac failure. As described in the results section, most patients with heart failure had cardiac adverse events (half of these patients had grade 3 cardiac events). Fortunately, exacerbation of symptoms was prevented by cardiac evaluation before anagrelide therapy, careful administration of anagrelide, and follow‐up of changes in brain natriuretic peptide. There have been rare reports concerning exacerbation of heart failure^26^; thus, careful follow‐up is needed for patients with heart disease.^27^ Although the incidences of thrombotic (15.1%; 3.7/100 patient years) and hemorrhagic events (7.5%; 1.8/100 patient years) were similar to, or slightly higher than, that in previous studies,^6^, ^8^, ^13^ these observed incidences might be attributed to the small sample size of our cohort. During both events, affected patients showed a high median WBC count and median neutrophil rate, suggesting a potential relationships between these factors and event onset.^28^ Three patients experienced MF transformation. The EXELS study found that time since diagnosis was a risk factors for MF transformation.^13^ In the present study, the time since diagnosis and time since initiation of anagrelide therapy were both relatively short. The first patient was diagnosed with a type 1 *CALR* mutation based on a high WBC count and high lactate dehydrogenase (LDH) level. The second patient had a high LDH level and splenomegaly. The third patient was diagnosed with type 1 *CALR* based on anemia and a high LDH level. These results may indicate a difficulty in differentiating between ET and prefibrotic primary myelofibrosis, rather than a causal relationship between adverse events and anagrelide therapy.

There have been a few studies regarding the efficacy of anagrelide and hydroxyurea combination therapy.^25^, ^29^ Most studies consisted of patients for whom anagrelide was added to hydroxyurea. The present study showed that favorable platelet counts could be achieved in patients for whom hydroxyurea was added to anagrelide, which suggested that the combination therapy provides good control of platelet count and tolerability. This might be because the combination of anagrelide and hydroxyurea enabled reduction of the daily doses of both drugs, thereby reducing the incidences of adverse events associated with each drug. Combination therapy may be useful for ET patients who have shown an insufficient response to anagrelide.

The findings of increased WBC count, neutrophil rate, and Hb level in the *JAK2*‐ET group in our cohort, compared with the *CALR*‐ET group, are consistent with previously reported findings.^30^, ^31^ In addition, the lack of a significant difference in platelet count between the two groups might be due to the small sample size of our cohort. There was no significant difference in the median daily dose of anagrelide between the two groups, and the rates of changes in WBC counts, Hb levels, and platelet counts from baseline were similar in both groups. Notably, the results were similar, in even among patients who received anagrelide monotherapy. Thus, the therapeutic effect of anagrelide is consistent, regardless of the genetic mutation profiles. Notably, the rate of PR was higher than that of CR in the *CALR*‐ET group. This suggests that attending physicians consider it is not necessary to achieve strict CR control in the *CALR*‐ET group, with respect to the low risk of thrombosis. Although Hb levels decreased in a similar manner, the incidence of anemia was significantly higher in the *CALR*‐ET group than in the *JAK2*‐ET group, because baseline Hb levels were significantly lower in the *CALR*‐ET group.

This study had several limitations. First, the patients in this study may have been younger and more motivated to undergo treatment than patients who received hydroxyurea as a first‐line therapy. However, the overall median age of the subjects in this study was 67.0 years, which suggests that anagrelide can be safely used as first‐line therapy in relatively in older people. Second, adverse events may have been underestimated because this was a retrospective study that relied on evaluations of medical records completed by attending physicians.

Hydroxyurea is widely used in cytoreduction therapy for ET patients and its efficacy has been demonstrated.^7^ Our study found that the use of anagrelide as a first‐line therapy for Japanese ET patients showed good thrombocytopenic effects and demonstrated a safety profile consistent with that of previous studies.^8^, ^9^, ^13^, ^22^, ^24^ THEs are closely related to driver gene mutations,^16^, ^32^ non‐driver mutations,^33^ WBC count,^28^ neutrophil rate,^34^ and other thrombotic risks, in addition to treatment choices. MF and AL are complications of ET, which can develop in patients who are not receiving cytoreductive treatment. Thus, it is impossible to fully identify medication‐related leukemogenesis.^12^ Therefore, it is important to develop a strategy that uses the benefits of both drugs: anagrelide‐based combination therapy may provide a good basis for future investigations of such strategies.

## CONFLICT OF INTEREST

Tomoki Ito and Yoshinori Hashimoto declare honoraria from Shire. The other authors declare that they have no conflict of interest.
