# Supplementary material for: Efficacy and safety of anagrelide as a first‐line drug in cytoreductive treatment‐naïve essential thrombocythemia patients in a real‐world setting
Source: Eur J Haematol. 2019 Jun 17;103(2):116–23. doi: 10.1111/ejh.13265 (PMC6851998; doi:10.1111/ejh.13265)
Supplement: Supplementary file 1 [file EJH-103-116-s001.docx]

**Suppl. Table 1** Changes of laboratory data in ET patients with *JAK2V617F* and *CALR* gene mutation

| Variable | | Mutation profiles | | *P* value |
| --- | --- | --- | --- | --- |
|  |  | *JAK2V617F* | *CALR* |  |
| before the  start of anagrelide | *N* | 34 | 11 |  |
|  | WBC, median; ×10^9^/L | 10.1 | 7.3 | 0.001 |
|  | Hb, median; g/dL | 14.2 | 13.6 | 0.040 |
|  | Plt, median; ×10^9^/L | 978 | 967 | 0.593 |
| 1 Mon after the start of anagrelide | *N* | 34 | 11 |  |
|  | WBC, median; ×10^9^/L | 10.6 | 7.0 | 0.001 |
|  | Hb, median; g/dL | 13.9 | 12.5 | 0.022 |
|  | Plt, median; ×10^9^/L | 757 | 613 | 0.947 |
| 2 Mon after the start of anagrelide | *N* | 34 | 11 |  |
|  | WBC, median; ×10^9^/L | 9.5 | 6.7 | 0.003 |
|  | Hb, median; g/dL | 13.5 | 12.6 | 0.028 |
|  | Plt, median; ×10^9^/L | 640 | 628 | 0.518 |
| 3 Mon after the start of anagrelide | *N* | 34 | 11 |  |
|  | WBC, median; ×10^9^/L | 10.0 | 6.8 | 0.001 |
|  | Hb, median; g/dL | 13.2 | 12.7 | 0.041 |
|  | Plt, median; ×10^9^/L | 583 | 555 | 0.558 |
| 6 Mon after the start of anagrelide | *N* | 33 | 11 |  |
|  | WBC, median; ×10^9^/L | 10.1 | 7.3 | 0.003 |
|  | Hb, median; g/dL | 13.2 | 12.1 | 0.232 |
|  | Plt, median; ×10^9^/L | 582 | 517 | 0.386 |
| 12 Mon after the start of anagrelide | *N* | 29 | 11 |  |
|  | WBC, median; ×10^9^/L | 9.8 | 7.4 | 0.044 |
|  | Hb, median; g/dL | 12.5 | 11.4 | 0.087 |
|  | Plt, median; ×10^9^/L | 590 | 525 | 0.785 |
| 24 Mon after the start of anagrelide | *N* | 22 | 7 |  |
|  | WBC, median; ×10^9^/L | 10.5 | 5.9 | 0.003 |
|  | Hb, median; g/dL | 13.0 | 10.4 | 0.056 |
|  | Plt, median; ×10^9^/L | 503 | 480 | 0.940 |
| 36 Mon after the start of anagrelide | *N* | 8 | 3 |  |
|  | WBC, median; ×10^9^/L | 10.3 | 6.1 | 0.012 |
|  | Hb, median; g/dL | 11.9 | 9.9 | 0.497 |
|  | Plt, median; ×10^9^/L | 455 | 481 | 0.630 |

*Non* month, *N* number of patients

**Suppl. Table 2** Change rate of laboratory data from baseline in ET patients with *JAK2V617F* and *CALR* gene mutation

| Variable | | Mutation profiles | | *P* value |
| --- | --- | --- | --- | --- |
|  |  | *JAK2V617F* | *CALR* |  |
| Change rate from baseline for 1 Mon after the start of anagrelide | *N* | 34 | 11 |  |
|  | WBC, median; % | +0.6 | -3.9 | 0.219 |
|  | Hb, median; % | -3.3 | -6.2 | 0.741 |
|  | Plt, median; % | -26.7 | -29.8 | 0.649 |
| Change rate from baseline for 2 Mon after the start of anagrelide | *N* | 34 | 11 |  |
|  | WBC, median; % | -5.8 | -7.0 | 0.822 |
|  | Hb, median; % | -5.4 | -7.0 | 0.194 |
|  | Plt, median; % | -40.2 | -31.2 | 0.866 |
| Change rate from baseline for 3 Mon after the start of anagrelide | *N* | 34 | 11 |  |
|  | WBC, median; % | -2.0 | -7.1 | 0.099 |
|  | Hb, median; % | -7.0 | -7.1 | 0.441 |
|  | Plt, median; % | -35.4 | -36.4 | 0.886 |
| Change rate from baseline for 6 Mon after the start of anagrelide | *N* | 33 | 11 |  |
|  | WBC, median; % | -1.2 | -10.0 | 0.538 |
|  | Hb, median; % | -11.8 | -10.1 | 0.714 |
|  | Plt, median; % | -42.0 | -39.8 | 0.669 |
| Change rate from baseline for 12 Mon after the start of anagrelide | *N* | 29 | 11 |  |
|  | WBC, median; % | -5.0 | -11.4 | 0.720 |
|  | Hb, median; % | -11.0 | -13.2 | 0.467 |
|  | Plt, median; % | -43.4 | -34.8 | 0.765 |
| Change rate from baseline for 24 Mon after the start of anagrelide | *N* | 22 | 7 |  |
|  | WBC, median; % | -14.0 | -25.3 | 0.070 |
|  | Hb, median; % | -10.7 | -12.2 | 0.328 |
|  | Plt, median; % | -46.7 | -51.0 | 0.784 |
| Change rate from baseline for 36 Mon after the start of anagrelide | *N* | 8 | 3 |  |
|  | WBC, median; % | +3.7 | -21.2 | 0.133 |
|  | Hb, median; % | -19.5 | -23.3 | 0.630 |
|  | Plt, median; % | -35.5 | -50.3 | 0.921 |

*Non* month, *N* number of patients

**Suppl. Table 3** Change rate of laboratory data from baseline in ET patients with *JAK2V617F* and *CALR* gene mutation received anagrelide monotherapy

| Variable | | Mutation profiles | | *P* value |
| --- | --- | --- | --- | --- |
|  |  | *JAK2V617F* | *CALR* |  |
| Change rate from baseline for 1 Mon after the start of anagrelide | *N* | 20 | 9 |  |
|  | WBC, median; % | -0.8 | -4.3 | 0.070 |
|  | Hb, median; % | -3.2 | -4.3 | 0.945 |
|  | Plt, median; % | -34.7 | -34.4 | 0.694 |
| Change rate from baseline for 2 Mon after the start of anagrelide | *N* | 20 | 9 |  |
|  | WBC, median; % | -5.6 | -8.1 | 0.365 |
|  | Hb, median; % | -6.1 | -7.0 | 0.417 |
|  | Plt, median; % | -46.5 | -36.6 | 0.562 |
| Change rate from baseline for 3 Mon after the start of anagrelide | *N* | 20 | 9 |  |
|  | WBC, median; % | -0.4 | -7.1 | 0.018 |
|  | Hb, median; % | -7.6 | -7.0 | 0.799 |
|  | Plt, median; % | -46.3 | -36.9 | 0.799 |
| Change rate from baseline for 6 Mon after the start of anagrelide | *N* | 19 | 9 |  |
|  | WBC, median; % | 0 | -10.0 | 0.629 |
|  | Hb, median; % | -11.8 | -7.0 | 0.417 |
|  | Plt, median; % | -45.6 | -39.8 | 1 |
| Change rate from baseline for 12 Mon after the start of anagrelide | *N* | 18 | 9 |  |
|  | WBC, median; % | -2.3 | -11.4 | 0.275 |
|  | Hb, median; % | -9.9 | -13.2 | 0.699 |
|  | Plt, median; % | -46.2 | -34.7 | 0.705 |
| Change rate from baseline for 24 Mon after the start of anagrelide | *N* | 13 | 6 |  |
|  | WBC, median; % | -8.2 | -21.2 | 0.059 |
|  | Hb, median; % | -9.8 | -20.7 | 0.336 |
|  | Plt, median; % | -48.7 | -39.2 | 0.503 |
| Change rate from baseline for 36 Mon after the start of anagrelide | *N* | 5 | 4 |  |
|  | WBC, median; % | -10.1 | -21.2 | 0.143 |
|  | Hb, median; % | -16.8 | -23.3 | 0.786 |
|  | Plt, median; % | -60.6 | -50.3 | 1 |

*Non* month, *N* number of patients
